# Shelf Life Extension and Nutritional Quality Preservation of Sour Cherries through High Pressure Processing

**DOI:** 10.3390/foods12020342

**Published:** 2023-01-11

**Authors:** Maria Concetta Tenuta, Elisa Artoni, Patrizia Fava, Cristina Bignami, Fabio Licciardello

**Affiliations:** 1 Department of Life Sciences, University of Modena and Reggio Emilia, 42122 Reggio Emilia, Italy; 2 Faculty of Science and Technology, Free University of Bozen-Bolzano, 39100 Bolzano, Italy; 3 Interdepartmental Research Centre for the Improvement of Agri-Food Biological Resources (BIOGEST-SITEIA), University of Modena and Reggio Emilia, 42122 Reggio Emilia, Italy

**Keywords:** bioactive compounds, microbial stability, HPP, quality preservation, sour cherries

## Abstract

The present study assessed the effectiveness of high pressure processing (HPP) for the quality maintenance of pitted sour cherries, with special regard to microbial stabilization and the maintenance of color and of chemical–nutritional properties. The HPP treatment (600 MPa for 3 min at 4 °C) was effective at minimizing the initial microbial load, which remained at negligible levels throughout 5 months of refrigerated storage. The color and total phytochemical content of sour cherries were not influenced by the HPP treatment and were maintained at levels comparable with the fresh product for 3 months of refrigerated storage. For longer storage periods, the typical red color decreased, in agreement with the content of total anthocyanins, which showed a significant decrease (up to 65% after 5 months). The antioxidant activity, measured by the ABTS and DPPH assays, was not affected by the HPP treatment, but slightly reduced during refrigerated storage. The study suggests that HPP may be exploited to extend the shelf life, while maintaining the fresh-like features of sour cherries, thus offering an alternative option to current preservation techniques (based on freezing or heating) commonly applied to this product.

## 1. Introduction

In the last few years, the demand for new technologies able to preserve and improve food quality has continuously increased. High pressure processing (HPP) is a non-thermal technology based on the application of high hydrostatic pressures (typically, from 100 to 600 MPa) for a short time to products contained in flexible packages, offering the potential to inactivate the vegetative forms of microorganisms through membrane disruption, thus maintaining fresh-like features [1,2,3]. The technology is very effective at preserving flavor, color, and nutritional properties of fruit and vegetable products, which gain a longer shelf life. Pressures comprised between 300 and 600 MPa inactivate molds, yeasts, and vegetative cells, the effectiveness being amplified by acidity [4]; on the other hand, bacterial spores require much higher pressures (up to 1200 MPa) for their inactivation, being the most baro-resistant microbial structures [5]. In addition, some enzymes are not completely inactivated by HPP and may cause textural and sensory changes, thus limiting the shelf life of HPP products to some weeks or months in refrigerated conditions. For instance, HPP-resistant enzymes are pectate lyase, pectate peroxidase, polygalacturonase, polyphenol oxidase, lipoxygenase, and pectin methylesterase [6].

Some of the fruit and vegetable products which have been effectively treated by HPP are apple, orange, lemon, and strawberry juices; tomato, raspberry, and guava purees; strawberry jam; lychee, kiwifruit, peaches, pears, and melon; avocado paste, fresh cut pineapple, red and white grape musts, onion, broccoli, cabbage, and carrots [7].

Sour cherries (*Prunus cerasus*), unlike sweet cherries, are mainly used as processed products and not for fresh consumption due to their high acidity, as well as the small fruit size of the most frequently cultivated varieties [8]. Sour cherry fruits are very perishable due to the high respiration and transpiration rates, and to the susceptibility to microbial spoilage that reduces postharvest life [9]. For these reasons, sour cherries are considered an industrial type of fruit [10,11,12]. Nearly 1,500,000 tons of sour cherries are produced worldwide, with the Russian Federation, Turkey, Ukraine, Serbia, and Poland as leading producers [13]. Over 60% of world production is supplied by Europe, where the cultivation is concentrated in eastern, southeastern, and central areas. Poland, Hungary, Romania, and Germany together contribute around 90% of the total EU production. This crop also includes significant niche productions, such as the “Amarena Brusca di Modena”, in the territory of Modena, Italy. These sour cherries are processed into the “Confettura di Amarene Brusche di Modena” which was the first jam to receive the European “Protected Geographical Indication” (P.G.I.) recognition and designation of origin [14]. Sour cherries are used for a series of processed products, such as canned fruits, jam and jellies, juice, syrups, spirits, bakery and confectionery products, and dried fruits [15,16]. However, some of the new cultivars have fruit characteristics also suitable for fresh consumption [8].

Recently, these fruits have attracted interest from the research viewpoint due to their high bioactive compound content, linked to their beneficial health properties. These were associated to the high content of anthocyanins and other polyphenols, mainly flavan-3-ols, and hydroxycinnamic acids [12,17,18,19,20,21]. The consumption of sour cherries, even in the processed form, has been linked to health benefits, such as the prevention of cardiovascular diseases through inhibition of lipid peroxidation [22], improvement of cognitive function, anti-inflammatory activity, and the ability to prevent and reduce oxidative stress [23,24]. A sour cherry-enriched diet decreased blood glucose levels preventing the onset of type 2 diabetes [25,26]. Moreover, diet integration with sour cherries has been proved to improve muscle damage recovery from physical exercise [11,27].

Alternative, non-thermal processing technologies such as pulsed electric fields (PEF) have been found to be effective for the inactivation of spores and vegetative cells of many pathogens in sour cherry juice [28]; however, no study has investigated the effectiveness of HPP for sour cherry preservation.

In the present study, we assessed the effectiveness of a HPP treatment for the microbial stabilization and quality maintenance of stoned sour cherries, through monitoring of color, pH, antioxidant activity, and phytochemical content, with special regard for polyphenols, flavonoids, and anthocyanins during refrigerated storage.

## 2. Materials and Methods

### 2.1. Sample

Pitted sour cherries (cv Amarena del Rio) were purchased from a local producer in the area of Modena, Italy, in June 2021. The orchard was planted in 2015 and trained at low vase with a planting distance of 4 × 2 m. At the stage of complete ripeness, fruits had an average (*n* = 40) weight of 9 g and a diameter of 25.5 mm; the total soluble content was 15.78 ± 1.88° Brix, and acidity was 22.15 ± 1.37 g/L. Fruits were randomly harvested at maturity stage and were mechanically pitted at the producer’s facilities. Pitted sour cherries were kept refrigerated and immediately transferred to the laboratory, where they were vacuum-packed (250 g) in pre-formed barrier bags.

### 2.2. High Pressure Processing (HPP)

Sample bags of pitted sour cherries were processed at HPP Italia s.r.l. (Traversetolo, Parma, Italy). A QFP 350L-600 equipment (Avure Technologies Inc., Kent, WA, USA) supplied with a 350 L hyperbaric chamber was used for the treatment carried out at 600 MPa for 3 min at 4 °C. The pressurized and control (untreated) samples were stored at refrigerated conditions (4 ± 1 °C) and sampled at 10-day intervals for the first month, then every 30 days until 5 months from treatment.

### 2.3. Microbiological Analysis

Microbial counts were determined before and immediately after the HPP treatment, and during refrigerated storage. Sour cherries (10 g) were homogenized in Stomacker with 90 mL sterile saline solution (0.9% NaCl) and suitably diluted before plating 1 mL in the following culture media: Plate Count Agar (PCA; Biolife, Milan, Italy) for total mesophilic aerobic bacteria, and Sabouraud Dextrose Agar (SDA; Biolife, Milan, Italy) for yeast and molds. Colonies were counted after incubation at 30 °C for 48 h for PCA and after incubation at 20 °C for 48 h for SDA; the results were expressed as colony-forming units per g of sample (CFU/g).

### 2.4. Colorimetric Analysis and pH Determination

The measurements of color parameters and pH were performed in triplicate on the homogenate used for the microbiological analysis. A Konica Minolta colorimeter model CM-700d/600d with D65 illuminant and 10° observer angle was used, which was provided with SpectraMagic NX software for the determination of CIELAB color parameters. In this color space, L* indicates the brightness, a* and b* the chromaticity coordinates: +a* is the direction of red, −a* is the direction of green, +b* is the direction of yellow and −b* is the direction of blue. The pH was measured by a Crison MicropH 2002 pH-meter (Crison Instruments S.A., Milan, Italy), previously calibrated with buffer solutions.

### 2.5. Sour Cherries Extraction Procedure

Untreated and HPP-treated sour cherries were extracted for the quantification of bioactive molecules, as previously described by Escribano-Bailon et al. [29] and Santos-Buelga et al. [30]. Briefly, 37.5 g of sour cherries were cut and extracted with 100 mL methanol: HCl 95:5 (*v*/*v*) (Carlo Erba, Milan, Italy) in an ultrasound-assisted maceration process with Sonomatic Ultrasonics Cleaners (150 W, 40 kHz frequency; Langford Electronics Ltd., Coventry, UK) for 20 min at room temperature. Afterwards, the solution was centrifuged for 10 min at 6000× *g*. The residue obtained was re-extracted two more times under the same conditions. The three extracts were combined, and a few mL of distilled water were added. Afterwards, the extracts were concentrated in a rotary evaporator to remove methanol.

### 2.6. Evaluation of Total Phenols, Flavonoids and Anthocyanins

Total phenols were quantified using the Folin–Ciocalteu method, previously described by Tenuta et al. [31]. The absorbance was read at 765 nm using a VWR UV-6300PC UV/Vis spectrophotometer. The results were expressed as mg of gallic acid equivalents/g of fruit.

The total flavonoid content was measured using the aluminum chloride colorimetric assay, previously described by Tenuta et al. [31]. The absorbance was read at 510 nm using a VWR UV-6300PC UV/Vis spectrophotometer and results were expressed as mg of quercetin equivalents/g of fruit.

The total anthocyanin content was quantified using the differential pH method [32]. The absorbance was read at 510 nm and 700 nm, in buffers at pH 1.0 and pH 4.5, using a VWR UV-6300PC UV/Vis spectrophotometer. The results were expressed as mg of cyanidine-3-*O*-glucoside equivalent/g of fruit, using a molar extinction coefficient (ε) of 26,900, molecular weight (MW) of 449.2, and an absorbance:

A = [(A_510_ − A_700_)_pH 1.0_ − (A_510_ − A_700_)_pH 4.5_].

The concentration of monomeric anthocyanin was calculated as:

(A × MW × dilution factor × 1000)/(ε × 1).

All analyses were conducted in triplicate.

### 2.7. Evaluation of Radical Scavenging Activity

Radical scavenging activity was examined using 2,2′-azino-bis (3-ethylbenzothiazoline-6-sulphonic acid) (ABTS) and 2,2-diphenyl-1-picrylhydrazyl (DPPH) assays, according to Tenuta et al. [31]. Briefly, a solution of ABTS radical was diluted to give an absorbance of 0.70 at 734 nm. This solution was mixed with the sour cherry extracts and after 5 min of incubation the absorbance was measured. ABTS^+^ radical scavenging activity was expressed as percent inhibition (I%) and was calculated as follows:

[(A_0_ − A)/A_0_] × 100

where A_0_ is the absorbance of the control reaction and A is the absorbance in the presence of the extract.

A DPPH solution (1.0 × 10^−4^ M) was mixed with the sour cherry extracts and left for 30 min in the dark. The absorbance was read at 517 nm. DPPH radical scavenging activity was expressed as percent inhibition (I%) and was calculated as follows:

[(A_0_ − A_1_)/A_0_] × 100

where A_0_ is the absorbance of the control and A_1_ is the absorbance in the presence of the extract.

For both assays, ascorbic acid was used as positive control.

### 2.8. Statistical Analysis

Statistical analysis was performed by one-way analysis of variance (ANOVA) followed by Tukey’s multiple range test (*p* < 0.05) using the SPSS statistical software (SPSS 20 for Windows; SPSS Inc., IBM, New York, NY, USA). The results were expressed as mean ± standard deviation.

## 3. Results

Treatments with HPP can be considered as a cold pasteurization and can be applied to fresh food products to make them microbiologically stable during refrigerated storage, avoiding a traditional heat treatment that could alter their sensory and nutritional characteristics. In the present study, pitted sour cherries were HPP-treated. Few studies in the literature have evaluated the effect of HPP on cherry juice [33], but no report is available on the application of HPP to sour cherry fruits.

### 3.1. Microbiological Analysis

Microbiological analyses were conducted on pitted sour cherries before and after the HPP treatment (3 min at 600 MPa) to assess the effective microbial inactivation and stabilization. Table 1 reports the counts (CFU/g) for total mesophilic bacteria and fungi (yeasts and molds), before and after the HPP treatment.

Yeasts and molds were completely inactivated by the HPP treatment, since counts were always below the limit of detection throughout storage, while a few colonies were detected for mesophilic bacteria after 20 days of storage. Results are in line with previous findings on fruit juice, indicating initial microbial loads of about 104 CFU/mL and post-treatment values below 10 CFU/mL [34]. Similar results were obtained by Cubeddu et al. [35] for apple juice under the same processing conditions. However, no previous data are available on the application of HPP on sour cherries, except a study by Bayindirli et al. [33] who showed the effectiveness of HPP treatment of sour cherry juice at inactivating various pathogenic bacteria, such as *Escherichia coli*, *Staphylococcus aureus*, and *Salmonella enteritidis*. Using 350 MPa at 40 °C for 5 min, these pathogens were inactivated, while treatment at lower pressure did not ensure microbiological stability, even if considerable reductions were observed. Although it has been reported that the pressurization for 1–15 min at 300–600 MPa guarantees the inactivation of vegetative forms of microorganisms in foods [36], the application of higher pressures in this range (i.e., 600 MPa) is more effective and does not cause any undesirable change to these categories of products when compared to lower pressures.

### 3.2. Monitoring of Color Parameters and pH

HPP treatment does not usually affect the pigments responsible for color in fruits and vegetables [37], and this factor is very important for consumer preferences [38]. Nevertheless, in the HPP-treated products, a change in color can be observed during storage; this effect depends on the incomplete inactivation of enzymes that can trigger undesired reactions leading to quality loss [7].

**Figure 1:**
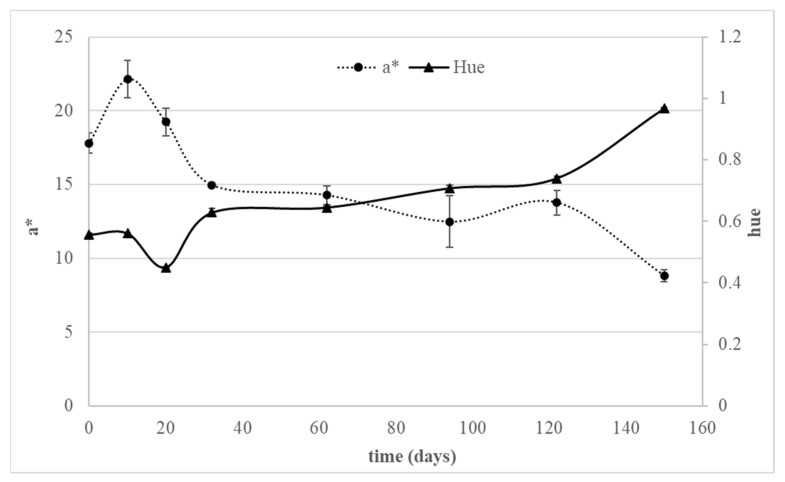
Color parameters (a* and hue) of HPP-treated sour cherries during refrigerated storage.

Color is commonly defined by instrumental parameters a* (green-red index), b* (blue-yellow index), hue, and saturation. As can be observed from Figure 1, the a* value shows a decrease during 5 months of refrigerated storage, which suggests a decrease in the characteristic red color note of the fresh product, while the b* coordinate did not show significant variations during storage time. The hue and saturation values were derived from a* and b* chromaticity coordinates. While saturation remained substantially stable, hue showed a slight increase ranging from about 0.4 to about 1. The observed color variations may arise from degradation reactions of anthocyanins, which are responsible for the red color of sour cherries. In the literature, different hypotheses are reported for anthocyanin degradation. The first one links anthocyanin stability with enzyme inactivation [39,40,41]. Peroxidase, polyphenol oxidase, and *β*-glucosidase are the enzymes involved in anthocyanin degradation during storage, hence their inactivation may result in a shelf-life extension. The second hypothesis regards the substrate specificity of enzymes acting on anthocyanins [39,42]. For example, the study of Zabetakis et al. [39] on strawberries showed low levels of pelargonidin 3-glucoside compared to pelargonidin 3-rutinoside and high activity of *β*-glucosidase, suggesting that *β*-glucosidase has a high specificity for pelargonidin 3-glucoside. Similar findings were obtained in strawberry jam treated with HPP [42]. The last hypothesis is linked to ascorbic acid presence: ascorbic acid may enhance the degradation of anthocyanins, even if it acts as an antioxidant agent [43]. In any case, anthocyanin degradation can be slowed down at refrigerated conditions.

The initial pH values (3.26), which have been reported to be synergistic with the HPP treatment, also create an unfavorable environment for the growth of microorganisms, thus ensuring stability. The monitoring of pH during refrigerated storage did not show significant changes (pH remained in the range 3.26–3.29), and this result agrees with the absence of microbial activity in the treated product.

### 3.3. Evaluation of Total Phenols, Flavonoids, and Anthocyanin Contents

Biological activities of fruits and vegetables depend on the bioactive compound content. Various classes of metabolites, in particular polyphenols, possess important bioactivities. Polyphenols represent the biggest group of plant secondary metabolites. They attract attention for the prevention of oxidative stress-related diseases [44]. Thus, the quantification of phytochemicals in plant foods is of paramount importance for understanding their health-promoting contribution in diets. In this study, the phytochemical content in total polyphenols, flavonoids, and anthocyanins was evaluated before and after the HPP treatment and during refrigerated storage.

Data (Table 2) show that the total polyphenol and anthocyanin content in non-processed sour cherries was 11.69 mg gallic acid equivalent (GAE)/g and 10.63 mg cyanidine-3-O-glucoside equivalent (CGE)/g, respectively; the total flavonoid content, expressed as quercetin equivalent (QE), was 1.32 mg/g. Based on data available in the literature, fresh sour cherries show a high content of polyphenols (179.5 mg GAE/100 g), flavonoids (37.5 mg QE/100 g), and anthocyanins (82.2 mg CGE/100 g) [16]. Moreover, Viljevac Vuletić et al. [45] reported values for polyphenols in the range of 4.87–13.73 mg GAE/g, and 2.02–6.42 mg CGE/g for anthocyanins.

**Figure 2:**
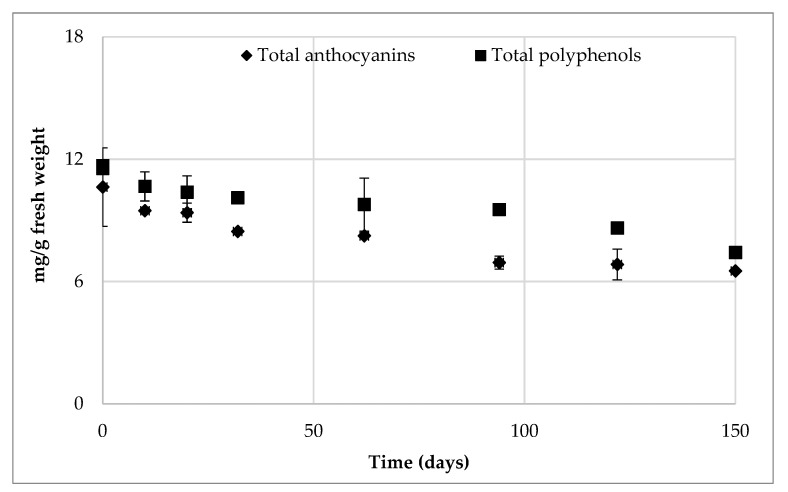
Total polyphenols and anthocyanins in HPP-treated sour cherries during refrigerated storage.

As can be inferred from the ANOVA results in Table 2, the total phytochemical content determined after HPP treatment, with particular regard for TP and TA, did not differ significantly from values determined on the fruits before processing, while some differences were observed during storage, and particularly in the last stage of the shelf-life tests. TP content showed a slight but not significant reduction with HPP processing, ranging between 11.69 and 11.54 mg GAE/g in the non-processed and HPP-treated samples, respectively. The total polyphenols decreased significantly during refrigerated storage, reaching 7.42 mg GAE/g after 5 months, showing an overall reduction of about 36%. Similarly, the TA content underwent a slight reduction with HPP processing, from 10.63 to 9.47 mg CGE/g in the unprocessed and HPP-treated samples, respectively, and a further degradation was observed throughout refrigerated storage, according to the trend observed for total polyphenols (Figure 2).

The TA loss accounted for about 30% (6.52 mg CGE/g) after 4 months and for about 65% (3.29 mg CGE/g) after 5 months. This significant loss in TA may explain the variation of color previously reported. A significant correlation (R = 0.9449, *p* ≤ 0.001) was observed between the TP and the TA content, and this underlines that anthocyanins make up most of the TP present in sour cherries.

Finally, the total flavonoid content showed a slight increase with HPP processing (1.75 mg QE/g) as compared to the control sample (1.32 mg QE/g), and a further increase during refrigerated storage by a maximum 30% after 3 months, followed by a slight decrease. Our results agree with the literature, confirming that HPP does not influence the total phytochemical content, which in fact can undergo significant changes during long storage. Compared to thermal treatments, HPP allows the preservation of the phytochemical content; in particular, a minimal effect on anthocyanin content has been reported [7]. Since anthocyanins are quite unstable, their content can be affected by high processing and storage temperatures. Ademović et al. [16] evaluated the influence of other processing techniques such as freeze-drying, vacuum concentration, and concentration on the bioactive content of sour cherries: fresh sour cherries possessed a high content in polyphenols, flavonoids, and anthocyanins with values of 179.5, 37.5, and 82.2 mg/100 g, respectively. All processing techniques tested, except concentration, preserved the contents in polyphenols and flavonoids, while all processing techniques significantly influenced the anthocyanin content, also reporting values 4.6 times lower than the fresh fruit. This result differs significantly from the present results, which show that HPP preserves all bioactives, including anthocyanins which are partially lost with other stabilization techniques.

### 3.4. Radical Scavenging Activity

**Figure 3:**
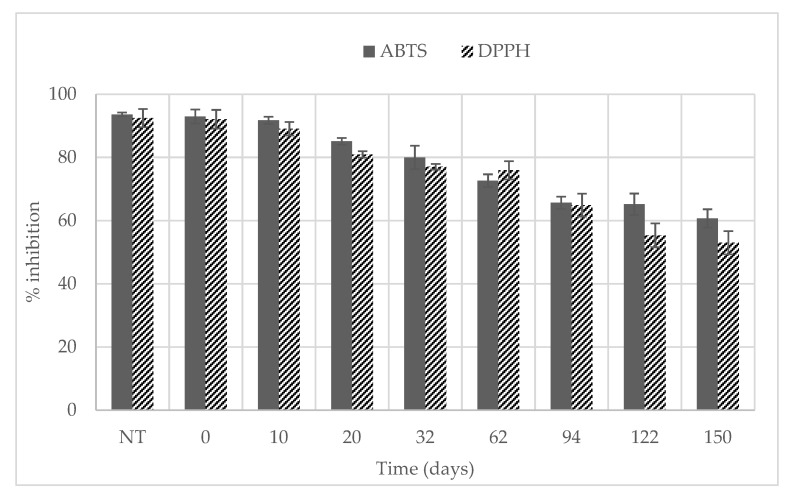
Radical scavenging activity of untreated and HPP-treated sour cherries during refrigerated storage.

In the present study, the radical scavenging activity of sour cherries was evaluated by the ABTS and the DPPH tests. Both assays are rapid, simple, and versatile for both lipophilic and hydrophilic antioxidant constituents, because they are compatible with non-polar and polar organic solvents [46]. The results of the radical scavenging activity of untreated and HPP-treated sour cherries have been expressed as % of inhibition (Figure 3).

The antioxidant capacity, measured by the capacity of a solution (sour cherry extracts) to directly react with radicals (ABTS or DPPH radicals), determining the reduction in absorbance, was not affected by HPP; in the ABTS test, the initial inhibition of the pressurized samples (92.95%) was similar to that of unprocessed fruits, while it decreased significantly throughout refrigerated storage, showing a progressive loss of antioxidant potential of the extracts (60.66% after 5 months). Similarly, the DPPH test showed no significant change of the antioxidant activity of sour cherries with HPP processing (92.42% and 92.07% for unprocessed and HPP-treated fruits, respectively) and a significant loss during refrigerated storage, accounting for nearly a 1.7-fold reduction in antioxidant activity after 5 months. The literature lacks studies on the antioxidant activity of sour cherries treated with HPP and, to the best of our knowledge, this is the first report on the radical scavenging capacities of HPP-treated sour cherries. Okur et al. [47] evaluated the total antioxidant activity of sour cherry pomace treated with different novel technologies (HPP, microwave-assisted extraction, and ultrasonic-assisted extraction). The use of HPP indicated a high antioxidant activity with 84.33% inhibition against DPPH radical; similar results were obtained with use of ultrasonic-assisted extraction (85.77%) and microwave-assisted extraction (89.9%). Ademović et al. [16] investigated the influence of other processing techniques (freeze-drying, vacuum concentration, and concentration) on the contents of bioactive compounds and on the antioxidant capacity of sour cherries. The highest antioxidant activity was attributed to fresh cherries with an IC_50_ value of 17.98 mg/mL in the DPPH test. Only one sample that presented less capacity to neutralize the DPPH radical was attributed to a cherry concentrate with an IC_50_ value of 64.32 mg/mL; instead, other samples tested presented IC_50_ values within the range 22.42–35.4 mg/mL.

## 4. Conclusions

In the last few years, the interest for innovative processing technologies that safeguard the nutritional and sensory quality of fresh products has increased: non-thermal alternatives to heat-based treatments such as HPP are being increasingly applied in the food industry with the potential to offer fresh-like, stable products. The present study evaluated the effectiveness of HPP treatment at stabilizing and maintaining quality parameters (microbiological, color, phytochemical content, and antioxidant activity) of pitted sour cherries. High pressure processing maintained the color, total polyphenols, and total anthocyanins unaltered when compared to the unprocessed fruits, with an almost complete inactivation of the spoilage microflora, which was guaranteed for at least 5 months under refrigerated conditions. The monitored parameters were maintained at high levels for at least 3 months of refrigerated storage; meanwhile, in the last part of the storage period, the characteristic red color of sour cherries showed a significant decrease, shifting to a yellow tone, according to a significant decrease (by about 65%) in total anthocyanins. HPP-treated sour cherries exerted antioxidant activity in a concentration-dependent manner; however, their activity gradually decreased during storage.

HPP is confirmed as a valuable alternative to conventional processing techniques, prolonging the availability of a fresh-like, additive-free product which is particularly appreciated in the production of confectionery and ice-creams, and which can also be exploited for fresh consumption thanks to its health-promoting effects.

## Figures and Tables

**Table 1:** Total mesophilic bacteria and yeast and mold counts (CFU/g) in raw and HPP-treated sour cherries during refrigerated storage.

	Time (Days)	Total Mesophilic Bacteria (CFU/g)	Yeasts and Molds (CFU/g)
Untreated sour cherries	0	2.4 × 10^5^	2.1 × 10^1^
	0	-	-
	10	-	-
	20	15	-
HPP-treated sour cherries	32	-	-
	62	-	-
	94	-	-
	122	-	-
	150	-	-

The sign “-” stands for colonies not detected.

**Table 2:** Total polyphenols (TP), flavonoids (TF), and anthocyanins (TA) in unprocessed and HPP-treated sour cherries.

	Time (Days)	TP (mg GAE/g)	TF (mg QE/g)	TA (mg CGE/g)
Control (untreated)	0	11.69 ± 0.24 ^a^	1.32 ± 0.05 ^e^	10.63 ± 1.93 ^a^
	0	11.54 ± 0.09 ^a^	1.75 ± 0.02 ^d^	9.47 ± 0.07 ^ab^
	10	10.67± 0.72 ^ab^	1.90 ± 0.01 ^c^	9.38 ± 0.47 ^ab^
	20	10.38 ± 0.81 ^ab^	1.90 ± 0.05 ^c^	8.46 ± 0.09 ^abc^
Sour cherries HPP-treated	32	10.11 ± 0.03 ^ab^	1.98 ± 0.01 ^c^	8.24 ± 0.07 ^abc^
	62	9.78 ± 1.30 ^ab^	2.18 ± 0.01 ^b^	6.93 ± 0.32 ^bc^
	94	9.53 ± 0.15 ^bc^	2.39 ± 0.04 ^a^	6.84 ± 0.75 ^bc^
	122	8.63 ± 0.04 ^bc^	2.22 ± 0.00 ^b^	6.52 ± 0.01 ^c^
	150	7.42 ± 0.02 ^c^	1.93 ± 0.02 ^c^	3.29 ± 0.00 ^d^

Different letters in columns indicate significant differences (*p* < 0.05) among mean values.

## Data Availability

The data presented in this study are available on request from the corresponding author.
